# 7-Meth­oxy-1-{[(*Z*)-2-nitro­phenyl­imino](phen­yl)meth­yl}-2-naphthol chloro­form monosolvate

**DOI:** 10.1107/S1600536810048002

**Published:** 2010-11-24

**Authors:** Atsushi Nagasawa, Akiko Okamoto, Noriyuki Yonezawa

**Affiliations:** a Department of Organic and Polymer Materials Chemistry, Tokyo University of Agriculture & Technology, 2-24-16 Naka-machi, Koganei, Tokyo 184-8588, Japan

## Abstract

In the title compound, C_24_H_18_N_2_O_4_·CHCl_3_, the phenyl and benzene rings make a dihedral angle of 38.60 (9)° and connect in an orientation almost perpendicular to the naphthalene ring system at dihedral angles of 78.73 (8) and 81.20 (7)°. The mol­ecule has a *Z* configuration about the C=N bond. In the crystal, mol­ecules are linked by inter­molecular O—H⋯N=C hydrogen bonds between the imino moiety and hy­droxy groups. Inter­molecular C—Cl⋯C inter­actions between Cl atoms of the CHCl_3_ mol­ecule and C atoms of the naphthalene rings are also present [Cl⋯C = 3.353 (2) and 3.326 (19) Å]. The nitro group and the chloro­form solvent mol­ecule are disordered over two positions with site occupancies of 0.884 (4) and 0.116 (4).

## Related literature

For the structures of closely related compounds, see: Mitsui *et al.* (2008 ▶); Nagasawa *et al.* (2010*a* ▶,*b* ▶,*c* ▶,*d* ▶).
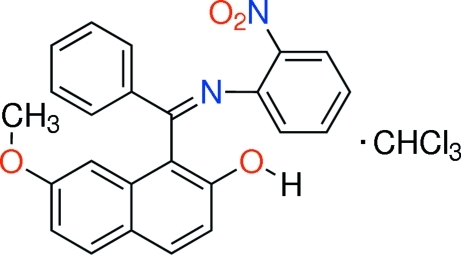


## Experimental

### 

#### Crystal data

C_24_H_18_N_2_O_4_·CHCl_3_
*M* *_r_* = 517.77
Monoclinic, 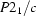

*a* = 13.2672 (6) Å
*b* = 11.2865 (6) Å
*c* = 17.2371 (9) Å
β = 109.114 (1)°
*V* = 2438.8 (2) Å^3^
*Z* = 4
Mo *K*α radiation
μ = 0.41 mm^−1^
*T* = 193 K
0.60 × 0.30 × 0.10 mm

#### Data collection

Rigaku R-AXIS RAPID diffractometer
Absorption correction: numerical (*NUMABS*; Higashi, 1999 ▶) *T*_min_ = 0.762, *T*_max_ = 0.960
38275 measured reflections
5576 independent reflections
4899 reflections with *I* > 2σ(*I*)
*R*_int_ = 0.024

#### Refinement

*R*[*F* ^2^ > 2σ(*F* ^2^)] = 0.041
*wR*(*F* ^2^) = 0.114
*S* = 1.06
5576 reflections
331 parameters
20 restraints
H-atom parameters constrained
Δρ_max_ = 0.50 e Å^−3^
Δρ_min_ = −0.57 e Å^−3^

### 

Data collection: *PROCESS-AUTO* (Rigaku, 1998 ▶); cell refinement: *PROCESS-AUTO*; data reduction: *CrystalStructure* (Rigaku/MSC, 2004 ▶); program(s) used to solve structure: *SIR2004* (Burla *et al.*, 2005 ▶); program(s) used to refine structure: *SHELXL97* (Sheldrick, 2008 ▶); molecular graphics: *ORTEPIII* (Burnett & Johnson, 1996 ▶); software used to prepare material for publication: *SHELXL97*.

## Supplementary Material

Crystal structure: contains datablocks global, I. DOI: 10.1107/S1600536810048002/pk2281sup1.cif

Structure factors: contains datablocks I. DOI: 10.1107/S1600536810048002/pk2281Isup2.hkl

Additional supplementary materials:  crystallographic information; 3D view; checkCIF report

## Figures and Tables

**Table 1:** Hydrogen-bond geometry (Å, °)

*D*—H⋯*A*	*D*—H	H⋯*A*	*D*⋯*A*	*D*—H⋯*A*
O1—H1⋯N1^i^	0.77	1.97	2.7160 (16)	163

Symmetry code: (i) 
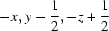
.

**Fig. 1.**
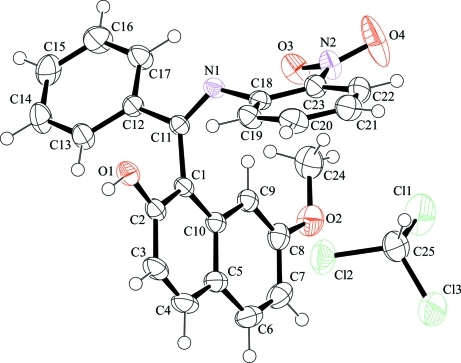
The asymmetric unit of compound (II) showing only the major component and atom labeling. Displacement ellipsoids are drawn at the 50% probability.

**Fig. 2.**
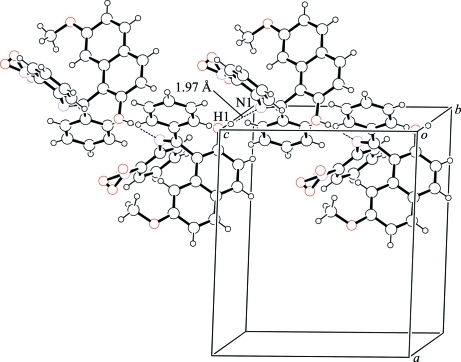
A partial crystal packing diagram of compound (II) (intermolecular O—H···N hydrogen bonds are shown as dashed lines).

**Fig. 3.**
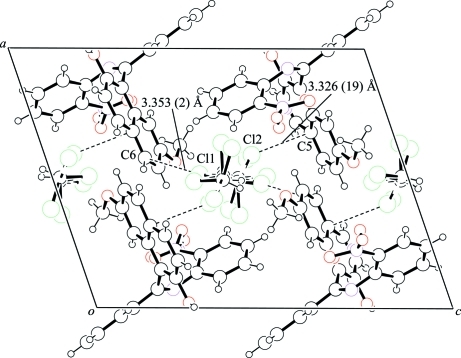
A partial crystal packing diagram of compound (II), viewed down the b axis (intermolecular Cl···C interactions are shown as dashed lines).

## supplementary crystallographic information

### Comment

Recently, we have reported the crystal structures of 1-monoaroylated naphthalene homologues having 2-hydroxy group exemplified by (4-chlorophenyl)(2-hydroxy-7-methoxynaphthalen-1-yl)methanone (Mitsui *et al*., 2008), (2-hydroxy-7-methoxynaphthalen-1-yl)(phenyl)methanone (Nagasawa *et al.*, 2010*a*) and (2-hydroxy-7-methoxynaphthalen-1-yl)(4-methylphenyl)methanone (Nagasawa *et al*., 2010*c*). The carbonyl group of these compounds are readily converted to the imino group by imination with aniline derivatives in the presence of TiCl_4 _and 1,4-diazabicyclo[2.2.2]octane (DABCO). The crystal structures of some of the imine compounds thus obtained have also revealed, e.g., 1-[(4-chlorophenyl)(phenylimino)methyl]-7-methoxy-2-naphthol-1,4-diazabicyclo[2.2.2]octane (2/1) (Nagasawa *et al.*, 2010*b*) and 7-methoxy-1-{[(*Z*)-2-nitrophenylimino](phenyl)methyl}-2-naphthol, (I) (Nagasawa *et al.*, 2010*d*). As a part of our ongoing studies on the synthesis and crystal structure analysis of triarylimine compounds, we prepared and analysed the crystal structure of the title compound (II), which is the regioisomer of (I).

An *ORTEPIII* (Burnett & Johnson, 1996) plot of (II) is shown in Fig. 1. In the molecule of (II), interplanar angles of the least-squares plane of the benzene ring (C18–C23) attached to nitrogen atom (N1) and benzene ring (C12–C17) attached to carbon atom (C11) of imine moiety against the naphthalene ring (C1–C10) are 81.20 (7) and 78.73 (8)°, respectively. The conformation of these groups resembles to that of (I). On the other hand, the interplanar angle between two benzene rings is 38.60 (9)°, which is smaller than that of (I), *i.e.* 87.15 (6)°. The molecule of (II) has a *Z* configuration for the imine vector.

In the crystal structure, the molecular packing of (II) is mainly stabilized by intermolecular hydrogen bond and van der Waals interaction. The intermolecular O—H···N hydrogen bond between the hydroxy and the imino groups on the naphthalene ring is observed [H1···N1 = 1.97 Å] (Fig. 2). In addition, one chloroform molecule and two aroylated naphthalene molecules are linked by Cl···C interactions along the *c* axis [Cl1···C6 = 3.353 (2) Å, Cl2···C5 = 3.326 (19) Å] (Fig. 3).

### Experimental

To a solution of (2-hydroxy-7-methoxynaphthalen-1-yl)(phenyl)methanone (0.2 mmol, 56 mg) in chlorobenzene (1 ml), a mixture of 2-nitroaniline (0.22 mmol, 30 mg), TiCl_4_ (0.33 mmol, 62.4 mg), DABCO (1.32 mmol, 148.0 mg) and chlorobenzene (1 ml) was added by portions at 363 K under nitrogen atmosphere. After the reaction mixture was stirred at 398 K for 1.5 h, the resulting solution was filtrated to remove the solid formed. The solvent was removed under reduced pressure to give crude material. The crude material thus obtained was subjected to crystalization from CHCl_3_/hexane to give compound (II) as yellow platelet (m.p. 453.0–454.0 K, yield 207 mg, 40%).

Spectroscopic Data: ^1^H NMR (300 MHz, DMSO-*d_6_*) δ; 10.25, (s, 1H), 7.85 (dd, *J* = 8.6, 1.4 Hz, 1H), 7.70–7.60 (m, 4H), 7.50–7.36 (m, 4H), 7.07–6.98 (m, 3H), 6.81 (dd, *J* = 8,6, 2.4 Hz, 1H), 6.70 (d, *J* = 2.4 Hz, 1H), 3.58 (s, 3H); ^13^C NMR (75 MHz, DMSO-*d_6_*) 166.8, 158.6, 154.0, 145.6, 140.6, 138.4, 134.5, 133.0, 131.9, 131.0, 130.4, 129.2, 128.8, 125.2, 124.9, 123.3, 120.8, 115.8, 115.2, 114.9, 102.2, 55.2; IR (KBr): 3427, 1622, 1602, 1515, 1341, 1210; HRMS (*m/z*): [*M* + H]^+^ calcd for C_24_H_19_N_2_O_4_, 399.1345; found, 399.1371.

### Refinement

All H atoms were introduced in calculated positions and treated as riding on their parent atoms with C—H = 1.00 Å (methine), 0.98 Å (methyl) or 0.95 Å (aromatic) with *U*_iso_(H) = 1.2 or 1.5*U*_eq_(C) and O—H = 0.77 Å with *U*_iso_(H) = 1.5*U*_eq_(O).

In the nitro group, N2/N2' and O3/O3' atoms were constrained to make the anisotropic displacement parameters equal. The distances between C23—N2 and C23—N2' were restrained to possess the same value within 0.020 standard deviation. Further restraints were applied to generate similar *U*_ij_ values within 0.010 standard deviation for the O4 and O4' atoms. N2'—O3' and N2'—O4' bond lengths and the angle were restrained to be similar within 0.020 standard deviation. The nitro groups of the *U*_ij_ in the direction of the bond were restrained to be equal within 0.010 standard deviation.

In the chloroform molecule, C25/C25', Cl1/Cl1', Cl2/Cl2' and Cl3/Cl3' were constrained to make the anisotropic displacement parameters equal. C25'—Cl1', Cl2' and Cl3' lengths and angles were restrained to be nearly equal within 0.020 standard deviation.

### Crystal data

**Table:**

C_24_H_18_N_2_O_4_·CHCl_3_	*F*(000) = 1064
*M**_r_* = 517.77	*D*_x_ = 1.410 Mg m^−^^3^
Monoclinic, *P*2_1_/*c*	Melting point = 453.0–454.0 K
Hall symbol: -P 2ybc	Mo *K*α radiation, λ = 0.71075 Å
*a* = 13.2672 (6) Å	Cell parameters from 30136 reflections
*b* = 11.2865 (6) Å	θ = 3.1–27.4°
*c* = 17.2371 (9) Å	µ = 0.41 mm^−^^1^
β = 109.114 (1)°	*T* = 193 K
*V* = 2438.8 (2) Å^3^	Platelet, colorless
*Z* = 4	0.60 × 0.30 × 0.10 mm

### Data collection

**Table:**

Rigaku R-AXIS RAPID diffractometer	5576 independent reflections
Radiation source: rotating anode	4899 reflections with *I* > 2σ(*I*)
graphite	*R*_int_ = 0.024
Detector resolution: 10.00 pixels mm^-1^	θ_max_ = 27.5°, θ_min_ = 3.1°
ω scans	*h* = −17→17
Absorption correction: numerical (*NUMABS*; Higashi, 1999)	*k* = −14→14
*T*_min_ = 0.762, *T*_max_ = 0.960	*l* = −22→22
38275 measured reflections	

### Refinement

**Table:**

Refinement on *F*^2^	Primary atom site location: structure-invariant direct methods
Least-squares matrix: full	Secondary atom site location: difference Fourier map
*R*[*F*^2^ > 2σ(*F*^2^)] = 0.041	Hydrogen site location: inferred from neighbouring sites
*wR*(*F*^2^) = 0.114	H-atom parameters constrained
*S* = 1.06	*w* = 1/[σ^2^(*F*_o_^2^) + (0.056*P*)^2^ + 1.0227*P*] where *P* = (*F*_o_^2^ + 2*F*_c_^2^)/3
5576 reflections	(Δ/σ)_max_ = 0.001
331 parameters	Δρ_max_ = 0.50 e Å^−^^3^
20 restraints	Δρ_min_ = −0.57 e Å^−^^3^

### Special details

**Table:**

Geometry. All e.s.d.'s (except the e.s.d. in the dihedral angle between two l.s. planes) are estimated using the full covariance matrix. The cell e.s.d.'s are taken into account individually in the estimation of e.s.d.'s in distances, angles and torsion angles; correlations between e.s.d.'s in cell parameters are only used when they are defined by crystal symmetry. An approximate (isotropic) treatment of cell e.s.d.'s is used for estimating e.s.d.'s involving l.s. planes.
Refinement. Refinement of *F*^2^ against ALL reflections. The weighted *R*-factor *wR* and goodness of fit *S* are based on *F*^2^, conventional *R*-factors *R* are based on *F*, with *F* set to zero for negative *F*^2^. The threshold expression of *F*^2^ > σ(*F*^2^) is used only for calculating *R*-factors(gt) *etc*. and is not relevant to the choice of reflections for refinement. *R*-factors based on *F*^2^ are statistically about twice as large as those based on *F*, and *R*- factors based on ALL data will be even larger.

### Fractional atomic coordinates and isotropic or equivalent isotropic displacement parameters (Å^2^)

**Table:**

	*x*	*y*	*z*	*U*_iso_*/*U*_eq_	Occ. (<1)
O1	0.02083 (8)	0.05421 (9)	0.26338 (7)	0.0340 (2)	
H1	0.0030	−0.0070	0.2736	0.051*	
O2	0.43281 (10)	0.33884 (12)	0.13788 (8)	0.0460 (3)	
N1	0.07278 (9)	0.34133 (10)	0.23258 (7)	0.0260 (2)	
N2	0.2246 (4)	0.5366 (4)	0.28500 (16)	0.0388 (7)	0.884 (4)
O3	0.1947 (6)	0.5115 (6)	0.21267 (17)	0.0434 (6)	0.884 (4)
O4	0.2636 (3)	0.6325 (2)	0.31061 (13)	0.0886 (10)	0.884 (4)
N2'	0.227 (4)	0.522 (4)	0.2773 (13)	0.0388 (7)	0.116 (4)
O3'	0.198 (5)	0.518 (6)	0.2029 (15)	0.0434 (6)	0.116 (4)
O4'	0.3015 (11)	0.5866 (14)	0.3138 (9)	0.046 (3)*	0.116 (4)
C1	0.14869 (10)	0.14673 (11)	0.21739 (8)	0.0244 (3)	
C2	0.11662 (11)	0.04855 (12)	0.25110 (8)	0.0284 (3)	
C3	0.18235 (13)	−0.05341 (12)	0.27254 (9)	0.0357 (3)	
H3	0.1592	−0.1208	0.2951	0.043*	
C4	0.27910 (13)	−0.05430 (13)	0.26056 (9)	0.0369 (3)	
H4	0.3223	−0.1233	0.2741	0.044*	
C5	0.31622 (12)	0.04563 (13)	0.22843 (8)	0.0310 (3)	
C6	0.41893 (13)	0.04875 (15)	0.21993 (9)	0.0391 (3)	
H6	0.4636	−0.0190	0.2348	0.047*	
C7	0.45494 (12)	0.14659 (17)	0.19098 (10)	0.0408 (4)	
H7	0.5243	0.1470	0.1862	0.049*	
C8	0.38870 (12)	0.24785 (14)	0.16805 (9)	0.0344 (3)	
C9	0.28882 (11)	0.24960 (12)	0.17571 (8)	0.0279 (3)	
H9	0.2454	0.3182	0.1604	0.033*	
C10	0.25066 (11)	0.14845 (11)	0.20667 (8)	0.0255 (3)	
C11	0.07401 (10)	0.24982 (11)	0.18894 (8)	0.0237 (2)	
C12	0.00079 (11)	0.25001 (12)	0.10229 (8)	0.0269 (3)	
C13	−0.03252 (13)	0.14463 (13)	0.05936 (9)	0.0361 (3)	
H13	−0.0093	0.0708	0.0856	0.043*	
C14	−0.09970 (15)	0.14713 (16)	−0.02191 (10)	0.0442 (4)	
H14	−0.1230	0.0749	−0.0505	0.053*	
C15	−0.13261 (14)	0.25356 (17)	−0.06123 (10)	0.0458 (4)	
H15	−0.1778	0.2546	−0.1169	0.055*	
C16	−0.09974 (16)	0.35897 (16)	−0.01956 (10)	0.0473 (4)	
H16	−0.1225	0.4324	−0.0466	0.057*	
C17	−0.03340 (14)	0.35763 (13)	0.06198 (9)	0.0380 (3)	
H17	−0.0112	0.4302	0.0904	0.046*	
C18	0.13766 (11)	0.35278 (12)	0.31576 (8)	0.0268 (3)	
C19	0.12082 (13)	0.27964 (13)	0.37559 (9)	0.0338 (3)	
H19	0.0736	0.2141	0.3595	0.041*	
C20	0.17225 (14)	0.30158 (15)	0.45846 (9)	0.0399 (4)	
H20	0.1600	0.2508	0.4984	0.048*	
C21	0.24114 (14)	0.39668 (15)	0.48345 (9)	0.0418 (4)	
H21	0.2768	0.4103	0.5402	0.050*	
C22	0.25782 (13)	0.47152 (15)	0.42562 (9)	0.0396 (3)	
H22	0.3043	0.5376	0.4424	0.047*	
C23	0.20625 (12)	0.44975 (13)	0.34255 (9)	0.0312 (3)	
C24	0.36876 (16)	0.44165 (17)	0.10900 (12)	0.0506 (4)	
H24A	0.4090	0.4993	0.0884	0.076*	
H24B	0.3495	0.4769	0.1542	0.076*	
H24C	0.3038	0.4192	0.0646	0.076*	
C25	0.4954 (2)	0.2617 (3)	0.4771 (2)	0.0463 (5)	0.884 (4)
H2	0.4638	0.2954	0.5176	0.056*	0.884 (4)
Cl1	0.51726 (10)	0.38033 (11)	0.41735 (8)	0.0718 (3)	0.884 (4)
Cl2	0.40495 (10)	0.15916 (14)	0.41674 (8)	0.0602 (3)	0.884 (4)
Cl3	0.61723 (8)	0.19467 (8)	0.53272 (8)	0.0620 (3)	0.884 (4)
C25'	0.5097 (15)	0.2618 (19)	0.4821 (15)	0.0463 (5)	0.116 (4)
H2'	0.4931	0.3008	0.5285	0.056*	0.116 (4)
Cl1'	0.4895 (8)	0.3540 (8)	0.3952 (6)	0.0718 (3)	0.116 (4)
Cl2'	0.4217 (9)	0.1492 (13)	0.4346 (7)	0.0602 (3)	0.116 (4)
Cl3'	0.6378 (6)	0.2033 (7)	0.5088 (5)	0.0620 (3)	0.116 (4)

### Atomic displacement parameters (Å^2^)

**Table:**

	*U*^11^	*U*^22^	*U*^33^	*U*^12^	*U*^13^	*U*^23^
O1	0.0372 (5)	0.0222 (5)	0.0436 (6)	−0.0058 (4)	0.0145 (4)	0.0038 (4)
O2	0.0392 (6)	0.0544 (7)	0.0499 (7)	−0.0097 (5)	0.0220 (5)	0.0005 (6)
N1	0.0309 (6)	0.0215 (5)	0.0245 (5)	0.0023 (4)	0.0073 (4)	0.0010 (4)
N2	0.0458 (8)	0.0336 (15)	0.0359 (8)	−0.0118 (11)	0.0118 (8)	−0.0040 (8)
O3	0.0662 (10)	0.0354 (11)	0.0318 (10)	−0.0064 (7)	0.0205 (12)	0.0025 (12)
O4	0.150 (3)	0.0520 (13)	0.0558 (11)	−0.0610 (16)	0.0229 (13)	−0.0100 (10)
N2'	0.0458 (8)	0.0336 (15)	0.0359 (8)	−0.0118 (11)	0.0118 (8)	−0.0040 (8)
O3'	0.0662 (10)	0.0354 (11)	0.0318 (10)	−0.0064 (7)	0.0205 (12)	0.0025 (12)
C1	0.0297 (6)	0.0191 (6)	0.0222 (6)	0.0007 (5)	0.0056 (5)	−0.0010 (4)
C2	0.0347 (7)	0.0210 (6)	0.0273 (6)	−0.0021 (5)	0.0072 (5)	−0.0005 (5)
C3	0.0485 (8)	0.0208 (6)	0.0347 (7)	0.0024 (6)	0.0095 (6)	0.0050 (5)
C4	0.0473 (8)	0.0263 (7)	0.0326 (7)	0.0121 (6)	0.0070 (6)	0.0028 (6)
C5	0.0354 (7)	0.0314 (7)	0.0232 (6)	0.0076 (6)	0.0054 (5)	−0.0026 (5)
C6	0.0362 (8)	0.0468 (9)	0.0312 (7)	0.0144 (7)	0.0070 (6)	−0.0022 (6)
C7	0.0295 (7)	0.0583 (10)	0.0343 (8)	0.0050 (7)	0.0099 (6)	−0.0063 (7)
C8	0.0338 (7)	0.0427 (8)	0.0271 (7)	−0.0055 (6)	0.0103 (6)	−0.0054 (6)
C9	0.0303 (6)	0.0286 (7)	0.0239 (6)	−0.0006 (5)	0.0077 (5)	−0.0027 (5)
C10	0.0300 (6)	0.0254 (6)	0.0188 (5)	0.0018 (5)	0.0048 (5)	−0.0028 (5)
C11	0.0263 (6)	0.0197 (6)	0.0254 (6)	−0.0012 (5)	0.0090 (5)	0.0018 (5)
C12	0.0285 (6)	0.0263 (6)	0.0249 (6)	0.0002 (5)	0.0074 (5)	0.0006 (5)
C13	0.0449 (8)	0.0281 (7)	0.0309 (7)	−0.0040 (6)	0.0066 (6)	−0.0008 (6)
C14	0.0521 (10)	0.0415 (9)	0.0323 (8)	−0.0111 (7)	0.0047 (7)	−0.0083 (7)
C15	0.0468 (9)	0.0559 (10)	0.0263 (7)	−0.0031 (8)	0.0005 (6)	0.0005 (7)
C16	0.0598 (11)	0.0414 (9)	0.0316 (8)	0.0084 (8)	0.0024 (7)	0.0088 (7)
C17	0.0505 (9)	0.0284 (7)	0.0295 (7)	0.0035 (6)	0.0057 (6)	0.0012 (6)
C18	0.0305 (6)	0.0241 (6)	0.0245 (6)	0.0051 (5)	0.0072 (5)	−0.0012 (5)
C19	0.0439 (8)	0.0267 (7)	0.0307 (7)	0.0011 (6)	0.0122 (6)	0.0006 (5)
C20	0.0564 (10)	0.0363 (8)	0.0274 (7)	0.0086 (7)	0.0141 (7)	0.0059 (6)
C21	0.0511 (9)	0.0434 (9)	0.0237 (7)	0.0078 (7)	0.0026 (6)	−0.0029 (6)
C22	0.0409 (8)	0.0378 (8)	0.0328 (8)	−0.0019 (6)	0.0023 (6)	−0.0059 (6)
C23	0.0341 (7)	0.0294 (7)	0.0282 (7)	0.0001 (5)	0.0077 (5)	0.0001 (5)
C24	0.0516 (10)	0.0490 (10)	0.0549 (10)	−0.0131 (8)	0.0225 (8)	0.0076 (8)
C25	0.0548 (12)	0.0498 (10)	0.0337 (9)	−0.0049 (9)	0.0137 (9)	−0.0057 (7)
Cl1	0.0736 (6)	0.0777 (5)	0.0555 (5)	−0.0211 (4)	0.0093 (4)	0.0162 (4)
Cl2	0.0497 (5)	0.0729 (5)	0.0506 (6)	−0.0098 (4)	0.0065 (4)	−0.0209 (5)
Cl3	0.0576 (4)	0.0652 (4)	0.0514 (5)	−0.0027 (3)	0.0017 (3)	−0.0042 (3)
C25'	0.0548 (12)	0.0498 (10)	0.0337 (9)	−0.0049 (9)	0.0137 (9)	−0.0057 (7)
Cl1'	0.0736 (6)	0.0777 (5)	0.0555 (5)	−0.0211 (4)	0.0093 (4)	0.0162 (4)
Cl2'	0.0497 (5)	0.0729 (5)	0.0506 (6)	−0.0098 (4)	0.0065 (4)	−0.0209 (5)
Cl3'	0.0576 (4)	0.0652 (4)	0.0514 (5)	−0.0027 (3)	0.0017 (3)	−0.0042 (3)

### Geometric parameters (Å, °)

**Table:**

O1—C2	1.3566 (18)	C12—C17	1.399 (2)
O1—H1	0.7695	C13—C14	1.393 (2)
O2—C8	1.3664 (19)	C13—H13	0.9500
O2—C24	1.428 (2)	C14—C15	1.378 (3)
N1—C11	1.2810 (17)	C14—H14	0.9500
N1—C18	1.4159 (17)	C15—C16	1.384 (3)
N2—O3	1.211 (3)	C15—H15	0.9500
N2—O4	1.219 (3)	C16—C17	1.393 (2)
N2—C23	1.470 (3)	C16—H16	0.9500
N2'—O3'	1.214 (17)	C17—H17	0.9500
N2'—O4'	1.230 (18)	C18—C19	1.395 (2)
N2'—C23	1.481 (17)	C18—C23	1.401 (2)
C1—C2	1.3814 (18)	C19—C20	1.389 (2)
C1—C10	1.4242 (18)	C19—H19	0.9500
C1—C11	1.5026 (17)	C20—C21	1.384 (3)
C2—C3	1.4178 (19)	C20—H20	0.9500
C3—C4	1.365 (2)	C21—C22	1.379 (2)
C3—H3	0.9500	C21—H21	0.9500
C4—C5	1.414 (2)	C22—C23	1.392 (2)
C4—H4	0.9500	C22—H22	0.9500
C5—C6	1.418 (2)	C24—H24A	0.9800
C5—C10	1.4249 (18)	C24—H24B	0.9800
C6—C7	1.361 (3)	C24—H24C	0.9800
C6—H6	0.9500	C25—Cl2	1.745 (3)
C7—C8	1.417 (2)	C25—Cl3	1.757 (3)
C7—H7	0.9500	C25—Cl1	1.770 (3)
C8—C9	1.374 (2)	C25—H2	1.0000
C9—C10	1.4217 (19)	C25'—Cl3'	1.739 (19)
C9—H9	0.9500	C25'—Cl2'	1.740 (18)
C11—C12	1.4915 (18)	C25'—Cl1'	1.770 (19)
C12—C13	1.394 (2)	C25'—H2'	1.0000
			
C2—O1—H1	111.6	C15—C14—H14	119.7
C8—O2—C24	117.60 (13)	C13—C14—H14	119.7
C11—N1—C18	123.21 (11)	C14—C15—C16	119.91 (14)
O3—N2—O4	122.5 (4)	C14—C15—H15	120.0
O3—N2—C23	117.9 (3)	C16—C15—H15	120.0
O4—N2—C23	119.5 (2)	C15—C16—C17	120.12 (15)
O3'—N2'—O4'	119 (3)	C15—C16—H16	119.9
O3'—N2'—C23	135 (3)	C17—C16—H16	119.9
O4'—N2'—C23	104.8 (17)	C16—C17—C12	120.37 (14)
C2—C1—C10	120.13 (12)	C16—C17—H17	119.8
C2—C1—C11	119.83 (12)	C12—C17—H17	119.8
C10—C1—C11	120.00 (11)	C19—C18—C23	117.54 (12)
O1—C2—C1	117.46 (12)	C19—C18—N1	120.21 (12)
O1—C2—C3	121.68 (12)	C23—C18—N1	121.25 (12)
C1—C2—C3	120.86 (13)	C20—C19—C18	120.74 (14)
C4—C3—C2	119.67 (13)	C20—C19—H19	119.6
C4—C3—H3	120.2	C18—C19—H19	119.6
C2—C3—H3	120.2	C21—C20—C19	120.69 (14)
C3—C4—C5	121.24 (13)	C21—C20—H20	119.7
C3—C4—H4	119.4	C19—C20—H20	119.7
C5—C4—H4	119.4	C22—C21—C20	119.75 (14)
C4—C5—C6	122.01 (13)	C22—C21—H21	120.1
C4—C5—C10	119.46 (13)	C20—C21—H21	120.1
C6—C5—C10	118.49 (14)	C21—C22—C23	119.63 (15)
C7—C6—C5	121.51 (14)	C21—C22—H22	120.2
C7—C6—H6	119.2	C23—C22—H22	120.2
C5—C6—H6	119.2	C22—C23—C18	121.62 (14)
C6—C7—C8	119.77 (14)	C22—C23—N2	116.26 (16)
C6—C7—H7	120.1	C18—C23—N2	122.07 (15)
C8—C7—H7	120.1	C22—C23—N2'	122.3 (9)
O2—C8—C9	124.92 (14)	C18—C23—N2'	115.9 (9)
O2—C8—C7	114.12 (14)	O2—C24—H24A	109.5
C9—C8—C7	120.96 (14)	O2—C24—H24B	109.5
C8—C9—C10	119.84 (13)	H24A—C24—H24B	109.5
C8—C9—H9	120.1	O2—C24—H24C	109.5
C10—C9—H9	120.1	H24A—C24—H24C	109.5
C9—C10—C1	121.99 (12)	H24B—C24—H24C	109.5
C9—C10—C5	119.41 (13)	Cl2—C25—Cl3	111.70 (19)
C1—C10—C5	118.60 (12)	Cl2—C25—Cl1	111.47 (19)
N1—C11—C12	117.23 (11)	Cl3—C25—Cl1	110.27 (16)
N1—C11—C1	124.51 (12)	Cl2—C25—H2	107.7
C12—C11—C1	118.13 (11)	Cl3—C25—H2	107.7
C13—C12—C17	118.83 (13)	Cl1—C25—H2	107.7
C13—C12—C11	121.31 (12)	Cl3'—C25'—Cl2'	107.2 (13)
C17—C12—C11	119.83 (12)	Cl3'—C25'—Cl1'	108.2 (13)
C14—C13—C12	120.25 (14)	Cl2'—C25'—Cl1'	98.0 (12)
C14—C13—H13	119.9	Cl3'—C25'—H2'	114.1
C12—C13—H13	119.9	Cl2'—C25'—H2'	114.1
C15—C14—C13	120.51 (15)	Cl1'—C25'—H2'	114.1
			
C10—C1—C2—O1	176.96 (11)	C1—C11—C12—C17	−151.40 (14)
C11—C1—C2—O1	−5.47 (18)	C17—C12—C13—C14	−0.8 (2)
C10—C1—C2—C3	−2.5 (2)	C11—C12—C13—C14	−179.07 (14)
C11—C1—C2—C3	175.10 (12)	C12—C13—C14—C15	1.1 (3)
O1—C2—C3—C4	−178.72 (13)	C13—C14—C15—C16	−0.7 (3)
C1—C2—C3—C4	0.7 (2)	C14—C15—C16—C17	0.1 (3)
C2—C3—C4—C5	1.2 (2)	C15—C16—C17—C12	0.2 (3)
C3—C4—C5—C6	176.36 (14)	C13—C12—C17—C16	0.2 (2)
C3—C4—C5—C10	−1.2 (2)	C11—C12—C17—C16	178.47 (15)
C4—C5—C6—C7	−178.21 (14)	C11—N1—C18—C19	−66.99 (18)
C10—C5—C6—C7	−0.6 (2)	C11—N1—C18—C23	124.71 (15)
C5—C6—C7—C8	−0.5 (2)	C23—C18—C19—C20	−1.3 (2)
C24—O2—C8—C9	−2.7 (2)	N1—C18—C19—C20	−170.00 (13)
C24—O2—C8—C7	176.52 (14)	C18—C19—C20—C21	0.2 (2)
C6—C7—C8—O2	−178.28 (14)	C19—C20—C21—C22	0.9 (3)
C6—C7—C8—C9	1.0 (2)	C20—C21—C22—C23	−0.9 (2)
O2—C8—C9—C10	178.85 (13)	C21—C22—C23—C18	−0.3 (2)
C7—C8—C9—C10	−0.3 (2)	C21—C22—C23—N2	177.4 (3)
C8—C9—C10—C1	178.66 (12)	C21—C22—C23—N2'	−176 (3)
C8—C9—C10—C5	−0.81 (19)	C19—C18—C23—C22	1.4 (2)
C2—C1—C10—C9	−177.09 (12)	N1—C18—C23—C22	169.96 (13)
C11—C1—C10—C9	5.34 (18)	C19—C18—C23—N2	−176.2 (3)
C2—C1—C10—C5	2.38 (18)	N1—C18—C23—N2	−7.6 (3)
C11—C1—C10—C5	−175.19 (11)	C19—C18—C23—N2'	177 (3)
C4—C5—C10—C9	178.91 (12)	N1—C18—C23—N2'	−14 (3)
C6—C5—C10—C9	1.27 (19)	O3—N2—C23—C22	168.9 (6)
C4—C5—C10—C1	−0.57 (19)	O4—N2—C23—C22	−14.9 (6)
C6—C5—C10—C1	−178.22 (12)	O3—N2—C23—C18	−13.4 (8)
C18—N1—C11—C12	179.69 (12)	O4—N2—C23—C18	162.8 (4)
C18—N1—C11—C1	−4.4 (2)	O3—N2—C23—N2'	33 (12)
C2—C1—C11—N1	94.25 (16)	O4—N2—C23—N2'	−151 (13)
C10—C1—C11—N1	−88.17 (16)	O3'—N2'—C23—C22	175 (7)
C2—C1—C11—C12	−89.89 (15)	O4'—N2'—C23—C22	7(5)
C10—C1—C11—C12	87.69 (15)	O3'—N2'—C23—C18	0(9)
N1—C11—C12—C13	−156.96 (14)	O4'—N2'—C23—C18	−168 (2)
C1—C11—C12—C13	26.88 (19)	O3'—N2'—C23—N2	−137 (20)
N1—C11—C12—C17	24.77 (19)	O4'—N2'—C23—N2	55 (10)

### Hydrogen-bond geometry (Å, °)

**Table:**

*D*—H···*A*	*D*—H	H···*A*	*D*···*A*	*D*—H···*A*
O1—H1···N1^i^	0.77	1.97	2.7160 (16)	163

Symmetry codes: (i) −*x*, *y*−1/2, −*z*+1/2.
